# Anatomy

**Published:** 1893-03

**Authors:** 


ANATOMY. A Manual for Students and Practitioners. By FRED. J. BROCKWAY, M. D., Assistant Demonstrator of Anatomy, College of Physicians and Surgeons, New York, and A. O'Malley, M. D., Instructor in Surgery, New York Polyclinic. Series edited by Bern B. Gallaudet, M. D., Demonstrator of Anatomy, College of Physicians and Surgeons, New York; Visiting Surgeon, Bellevue Hospital, New York. Philadelphia: Lea Brothers & Co.

The books, as a class, to which this one belongs are multiplying too rapidly, and the only reason for their existence seems to be to spread the author's name and fame rather than to really supply a deficiency. In justice, however, to the author of this quiz-book, it can be said that some useful additions have been made, such, for example, as the various tables of homologies, the excellent and detailed descriptions of the action of muscles, and the variations from the normal type that may occur, and the useful glossary at the termination of the book. The adoption of Henle's classification seems to us judicious.

While the book belongs to a class with which we are not in full sympathy, it is only fair to say that this one tends to elevate its class. The book is convenient in size, and is up to the same high standard that the publishers always give their readers. J. P.

